# Recurrent exercise-induced acute kidney injury associated with hypouricemia: a case report and literature review

**DOI:** 10.1186/s12882-023-03378-w

**Published:** 2023-12-21

**Authors:** Jie Zhou, Min Zhang, Qionghong Xie, Ningxin Xu, Mingxin Li, Ming Zhang, Chuanming Hao

**Affiliations:** 1 Division of Nephrology, Huashan Hospital, Fudan University, Shanghai, China; 2 Department of Nephrology, Shuguang Hospital, Shanghai University of Traditional Chinese Medicine, Shanghai, China

**Keywords:** Hereditary renal hypouricemia, Uric acid reabsorption, Exercise-induced acute kidney injury, *SLC2A9* gene

## Abstract

**Background:**

Hereditary renal hypouricemia (RHUC) is a heterogenous disorder characterized by defective uric acid (UA) reabsorption resulting in hypouricemia and increased fractional excretion of UA. RHUC is an important cause of exercise-induced acute kidney injury (EIAKI), nephrolithiasis and posterior reversible encephalopathy syndrome (PRES). We present here an unusual case of a patient with RHUC who presented with recurrent EIAKI and had two heterozygous mutations in the *SLC2A9* gene.

**Case presentation:**

A 43-year old man was admitted to our clinic because of bilateral loin pain, nausea and sleeplessness for 3 days after strenuous exercise. The laboratory results revealed increased levels of blood urea nitrogen (BUN) (15 mmol/l) and serum creatinine (Scr) (450 μmol/l), while the UA level was extremely low at 0.54 mg/dl, and his fractional excretion of urate (FE-UA) was 108%. The patient had an episode of acute kidney injury after playing soccer approximately 20 years ago, and on routine physical examination, his UA was less than 0.50 mg/dl. In view of the marked hypouricemia and high FE-UA, a diagnosis of RHUC was suspected, which led us to perform mutational screening of the *SLC22A12* and *SLC2A9* genes. DNA sequencing revealed no mutation in *SLC22A12* gene, but two heterozygous mutations in the *SLC2A9* gene.

**Conclusions:**

This is a rare report of a patient with RHUC2 due to the mutation of *SLC2A9*. And this unique symptom of EIAKI and decreased or normal serum concentrations of UA warrant more attention as an early cue of RHUC.

## Background

RHUC is a rare hereditary disease caused by impaired urate transport: reduced urate reabsorption and/or increased secretion [1, 2]. The incidence of renal hypouricemia has been reported to be 0.15% to 0.72% [3]. Two types of RHUC have been described. RHUC1 is caused by mutations in the *SLC22A12* gene(OMIN #220,150), while RHUC2 is caused by mutations in the *SLC2A9* gene(OMIN #612,076). More than 150 RHUC patients with mutations in the *SLC22A12* gene have been described all over the world, with the majority belonging to the Asian population. The highest reported frequencies far have been 2.30–2.37% in Japan and 0.4% in Korea, with the specific mutation sites identified as c.774G > A (p.W258X) and c.269G > A (p.R90H), respectively. However, RHUC1 patients have also been documented in other ethnic groups, including Arab, Iraqi Jews, Caucasians, and Roma, as well as in geographically non-contiguous countries. Notably, the c.1245_1253del and c.1400C > T variants have unexpectedly high prevalence ﻿of 1.92% and 5.56%, respectively, among the Roma population in five regions spanning the Czech Republic, Slovakia, and Spain. These findings highlight the uneven geographical and ethnic distribution of *SLC22A12* mutations, necessitating consideration in non-Asian patients [4, 5]. Whereas only approximately 20 cases of RHUC2 have been reported worldwide [6–8]. Patient ethnicity is diverse and includes Japanese, Chinese, Ashkenazi-Jewish, Anglo-Saxon, Greek, and Czech; to date, only few Caucasian families with a *SLC2A9* mutation have been reported [9]. RHUC2 exhibits a broader clinical variability compared to RHUC1 [10]: UA levels observed in heterozygous patients with *SLC2A9* mutations are similar to those with compound heterozygous and/or homozygous *SLC22A12* mutations. However, compound heterozygous and/or homozygous RHUC2 patients have significantly lower serum UA levels, approaching zero, and markedly higher renal excretion, exceeding 100% [2, 11]. On the other hand, heterozygous RHUC2 family relatives exhibit normal levels of UA [12, 13].

Here we report an unusual case of a patient with RHUC2 who presented with recurrent EIAKI and had two heterozygous mutations in the *SLC2A9* gene.

## Case presentation

A 43‐year‐old man was admitted because of bilateral loin pain, nausea and sleeplessness for 3 days after strenuous exercise, but without oliguria, hematuria or myalgia. There was no history of nephrolithiasis, and family history was unremarkable. He took regular antihypertensive drug amlodipine. He had no smoking history and wasn’t exposed to known chemicals or solvents. On admission, his blood pressure was 167/103 mmHg. The other physical examination was unremarkable.

The laboratory results revealed increased levels of BUN (15 mmol/l) and Scr (450 μmol/l), while the UA level was extremely low at 32 μmol/l(0.54 mg/dl) (normal range 3.50–8.50 mg/dl). His creatine kinase (CK), lactate dehydrogenase (LDH) and complete blood count were all normal. 24-h urinary protein was 0.39 g (normal range < 150 mg), urinary protein/creatinine ratio was 417 mg/g (normal range < 200 mg/g), urinary albumin/creatinine ratio was 260 mg/g (normal range < 30 mg/g), urine β2 microglobulin was 0.57 mg/l (normal range < 0.2 mg/l). The patient’s FE-UA was 108%. Urinalysis showed no glucose, red blood cells or casts. Serologic parameters were all within normal range, including complement, antinuclear antibody (ANA), double-stranded DNA (dsDNA), antineutrophil cytoplasmic antibody (ANCA), anti-glomerular basement membrane (GBM) antibodies, and hepatitis B and C antibody. His renal ultrasound showed normal-size kidneys with no evidence of obstruction (right:125*65 mm, left:115*65 mm).

Within one week, his renal function started to recover spontaneously, and urinalysis showed no protein. Based on the above results, no kidney biopsy was performed.

The patient recalled that he had an episode of acute kidney injury after playing soccer approximately 20 years ago. At that time, his Scr was up to 900 μmol/l, and returned to normal range within one month without dialysis.

On a previous admission, his Scr was 65 μmol/l, BUN was 5 mmol/l, UA was less than 30 μmol/l(0.50 mg/dl).

None of his family members had hypouricemia during routine biochemistry examination (including his parents and his sister).

In view of the marked hypouricemia and high FE-UA, a diagnosis of RHUC was suspected, which led us to perform mutational screening of the *SLC22A12* and *SLC2A9* genes. However, only the patient but not other family members received the genetic test per the patient’s and his sister’s request.

DNA sequencing revealed no mutation in SCL22A22 gene, but two heterozygous mutations in the *SLC2A9* gene.

**Fig. 1:**
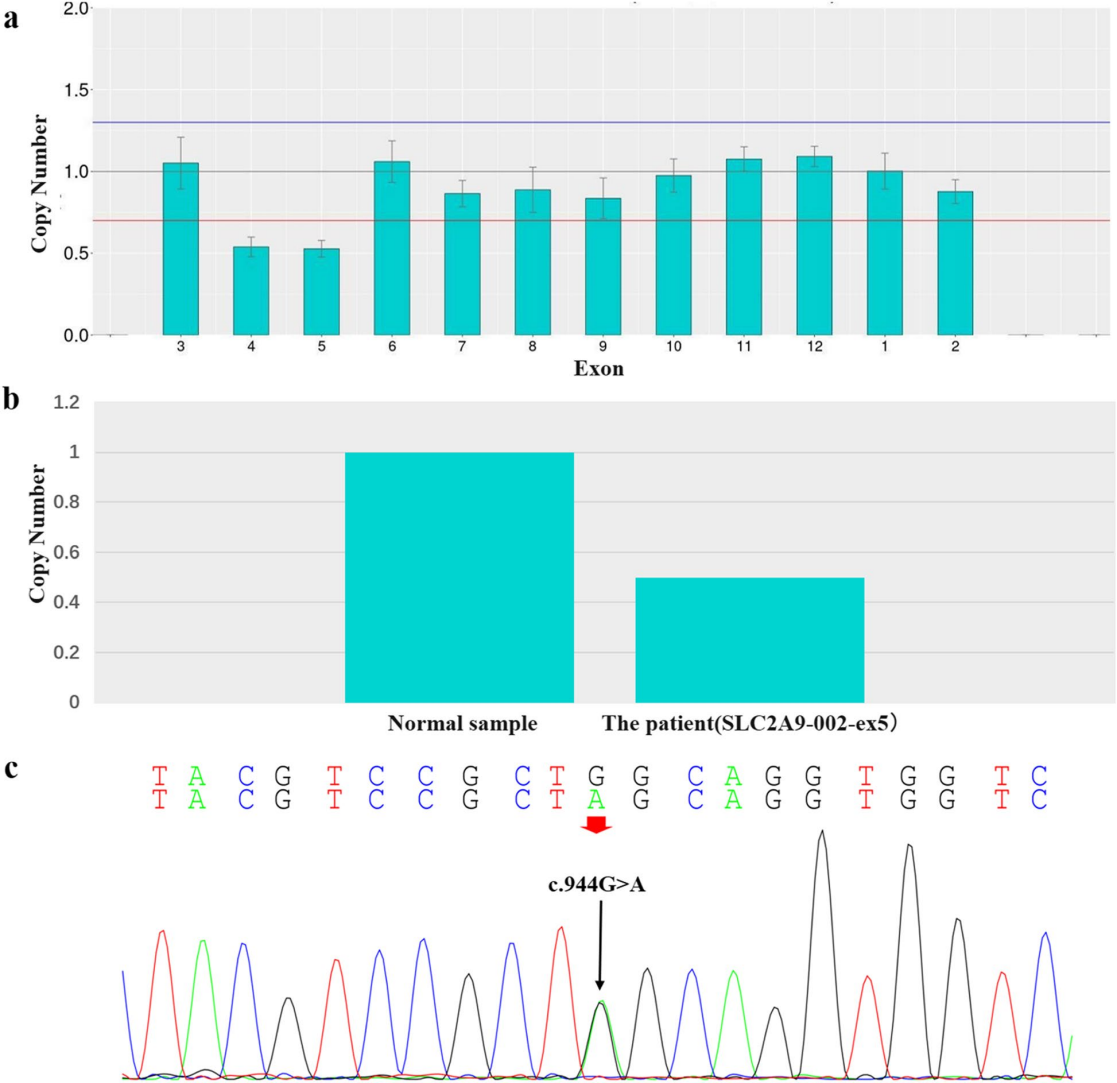
DNA sequencing for the mutations. The patient carried two heterogeneous mutations in *SLC2A9* gene. **a**. CNV analysis suggested a deletion of exon 4 and 5 in the *SLC2A9* gene. **b**. A length of 146 bp heterozygous deletion (chr4:9,982,216–9,982,361) in exon 5 of the *SLC2A9* gene was verified by qPCR-SYBR Green I method. **c**.A heterozygous nonsense mutation (c.944G > A, pTrp315Stop) in exon 7

Copy number variation(CNV) analysis of the next generation sequencing(NGS) data suggested a deletion of exon 4 and 5 in the *SLC2A9* gene. A length of 146 bp heterozygous deletion (chr4:9,982,216–9,982,361) in exon 5 of the *SLC2A9* gene was verified by qPCR-SYBR Green I method. Moreover, there was a heterozygous nonsense mutation (c.944G > A, pTrp315Stop) that introduced a premature “stop” codon located in exon 7 of the *SLC2A9* gene (Fig. 1). These mutations have not been reported previously.

**Fig. 2:**
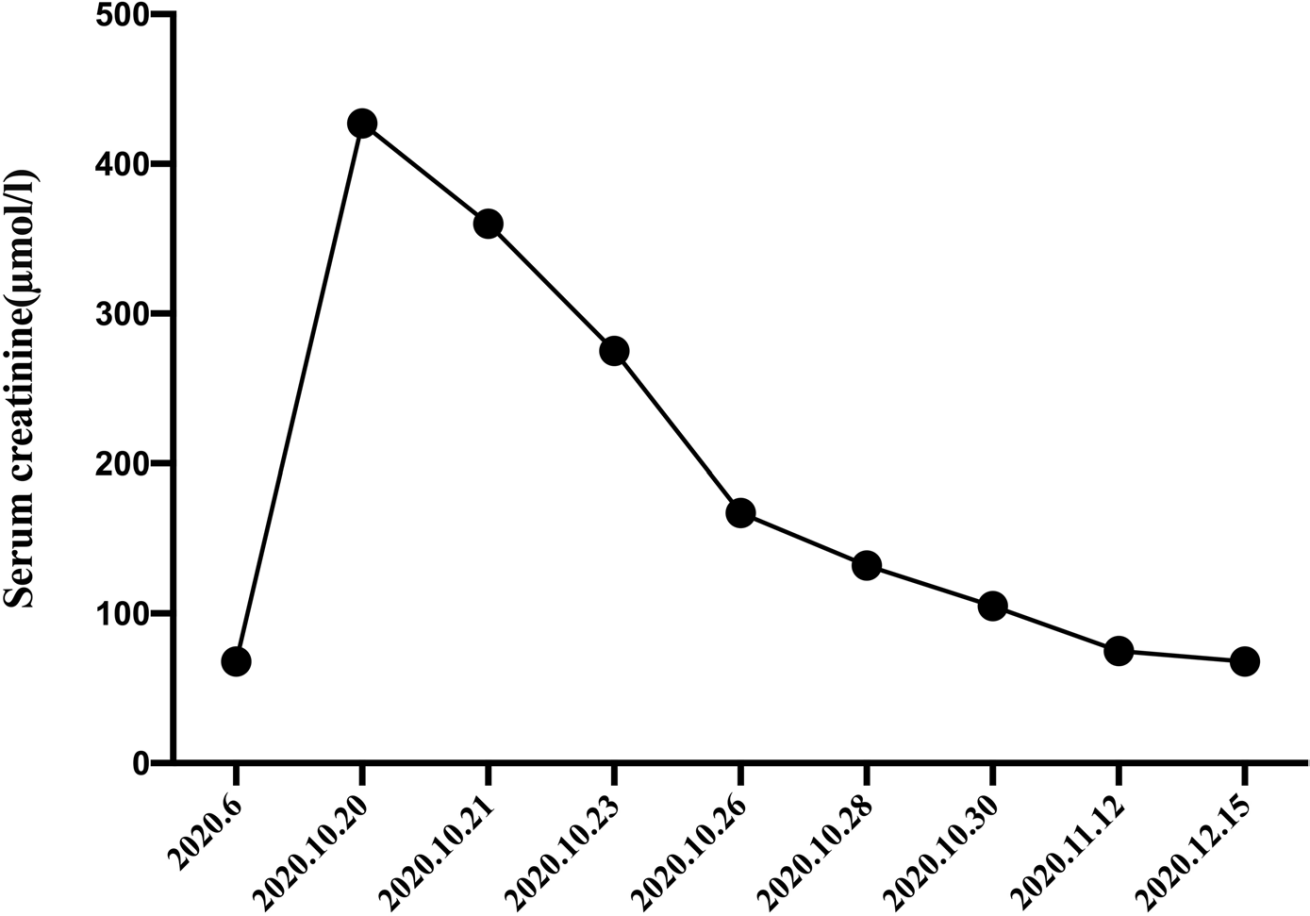
Evolution of serum creatinine

Two months after his discharge from the hospital, his renal function was improved remarkably (Scr recovered to 75 μmol/l), suggesting recovery of the kidneys from AKI (Fig. 2).

## Discussion and conclusion

There are two types of RHUC. RHUC1 is caused by mutations in the gene *SLC22A12* that encodes a renal urate-anion exchanger named urate transporter 1(URAT1) [1, 14]. URAT1 is the first identified renal urate-anion transporter, which is mainly expressed in the apical side of the proximal tubule, and is responsible for a large portion of proximal urate reabsorption [1]. RHUC2 is caused by mutations in the *SLC2A9* gene, which encodes a high-capacity glucose and urate transporter, named glucose transporter 9 (GLUT9) [15]. GLUT9 is expressed in human kidney proximal tubule epithelial cells. Two GLUT9 variants have been identified: the GLUT9S and GLUT9L [2, 8, 15, 16]. GLUT9L is localized to the basolateral side and GLUT9S to the apical side [17]. The GLUT9L has been suggested to be the only transporter that mediates the efflux of the urate from the cells across the basolateral membrane [11]. Whereas urate absorption from the tubular lumen into the cells is mediated by URAT1, GLUT9S and possibly other apical transporters [18].

Patients with homozygous mutations in *SLC22A12* typically have serum UA concentrations below 59.5 μmol/l(1.0 mg/dl) and a partial urate reabsorption defect with FE-UA between 40 and 90%. In contrast, the loss of function mutations in *SLC2A9* precludes all urate from exiting the cells from basal lateral membrane, thus completely blocking urate absorption. The net effect is that the value of FE-UA is greater than 100%, the values above 100% reflecting urate secretion. So loss-of-function mutations in *SLC2A9* cause more severe hypouricemia and render patients more vulnerable for complications, such as nephrolithiasis, EIAKI and PRES [9].

Nephrolithiasis and EIAKI are reported to have a prevalence of 8.5% and 6.5% in patients with RHUC based on mutational screening [11, 19]. PRES was only described in 3 patients with EIAKI due to RHUC till now [20–22]. Acute kidney disease associated with renal hypouricemia was first described in 1989 by Erley et al. [23].The relative risk was assessed in a study of 13 patients presenting with AKI following exercise, three of whom (23%) had RHUC [24]. A retrospective investigation described 54 patients’ clinical features of EIAKI associated with RHUC. The study revealed that the majority of patients were males, with a male: female ratio of 8:1. During follow-up, 13 patients (24%) developed recurrent AKI [25].

There is a difference in the occurrence rate of AKI between RHUC1 and RHUC2. In RHUC1, the loss of URAT1 function results in partial UA absorption defect. In RHUC2, the heterozygous GLUT9 mutation causes hypouricemia due to haploinsufficiency rather than a dominant negative effect. Homozygous GLUT9 mutations lead to severe hypouricemia through loss-of-function mutations and completely block the efflux of UA, preventing its absorption by all apical transporters, including URAT1. Therefore, reports indicate that heterozygous RHUC2 patients exhibit UA levels similar to those with URAT1 mutations, while homozygous and/or compound heterozygous patients have lower serum UA levels (close to 0 mg/dL) and higher FE-UA (> 100%), compared with 13% in those with heterozygous GLUT9 mutations and 40 to 90% in patients with homozygous loss of URAT1 [6, 11] (Table 1).

**Table 1:** Characterizations of RHUC

	RHUC1	RHUC2	
Mutated gene	*SLC22A12*	*SLC2A9*	
Mutated protein	URAT1	GLUT9	
Inheritance pattern	Autosomal recessive	Autosomal recessive	
Serum UA	0.5–1.0 mg/dl (Homozygous or compound heterozygous)	0.5–1.0 mg/dl (Heterozygous)	0–0.2 mg/dl (Homozygous or compound heterozygous)
FE-UA	40–90%	13%	> 100%
UA transport defect	Partial absorption defect	Partial absorption defect	Total reabsorption defect
Location (proximal tubules)	Apical side	Basolateral side and apical side	
Complications	Nephrolithiasis, EIAKI and PRES	Nephrolithiasis, EIAKI and PRES	

When AKI occurs in patients with RHUC after strenuous exercise, the initial symptoms are often abdominal pain and nausea/vomiting, usually occurring within 6–12 h after exercise. In addition, during the onset of AKI, the blood UA level may be close to normal due to kidney dysfunction, so it may not attract enough attention of clinicians. The prevention of AKI in RHUC includes avoiding strenuous exercise and ensuring sufficient fluid intake after exercise. Supportive treatment is important after AKI attack. The prognosis of RHUC-associated AKI is benign, only a few patients required hemodialysis, while recurrent AKI may lead to chronic kidney disease (CKD) in some patients [25, 26]. Therefore, early recognition and treatment are important to improve the renal outcome.

The mechanism by which RHUC causes EIAKI remains controversial. Firstly, oxidative stress from reactive oxygen species generated during exercise leads to renal vasoconstriction, ischemia, oxidant injury [27], resulting in reduced glomerular filtration rate and ATN; However, there were no differences observed in oxidative stress markers between RHUC patients and healthy participants before and after exercise [28]. Secondly, during exercise, increased UA production leads to UA excretion stress and precipitation in the tubules, as occurs in tumor lysis syndrome or UA nephropathy. Although Erley's description confirms the histological correlation between uric acid nephropathy and acute renal failure, further reports have not substantiated this finding. In fact, most kidney biopsies reveal acute tubular injury without intratubular UA deposition [23, 25, 29] **(**Table 2**)**.Thirdly, a study analyzed the purine metabolism in RHUC patients and found that, compared to healthy participants, RHUC patients exhibited a rapid decrease in blood xanthine concentration and a significant increase in urinary xanthine concentration after exercise. The decrease in blood xanthine, which serves as a salvage pathway substrate, can reduce ATP resynthesis, resulting in the loss of renal tubular ATP and EIAKI [28]. Fourthly, reduced clearance of urate-coupled anions as a result of loss-of-function mutations of either URAT1 or GLUT9 may exert toxic effects on renal proximal tubules, leading to toxic ATN [11]. Furthermore, in patients with EIAKI, it was observed through CT scans performed after the administration of iv contrast agents that there were patchy, wedge-shaped, and high-density areas in both kidneys due to delayed excretion of the contrast agent. This phenomenon may be attributed to transient renal segmental ischemia caused by renal vascular spasm. These findings suggest that renal vascular spasm during exercise may lead to a similar ischemia–reperfusion-like kidney injury following physical activity [24, 30].

**Table 2:** Histopathological manifestations of renal biopsy in RHUC-induced EIAKI

Case reports	Patients number	Pathological Description
Toshiyuki [25]	28	Acute tubular necrosis was observed in 22 patients, minimal change was present in 6 patients, and chronic renal lesions were identified in 1 patient
Erley [23]	1	Amorphous uric acid crystals in some of the tubular lumina and mild to moderate interstitial inflammation
Ishikawa [24]	3	The kidneys to be recovering from acute tubular necrosis; no evidence of urate deposition was noted
Numabe [31]	1	Acute tubular necrosis
Wang [32]	1	Acute tubular necrosis
Windpessl [9]	1	Subtle signs of acute tubular injury without intratubular UA precipitation

*SLC2A9* was first identified as a novel member of the facilitative glucose transporter family in 2000 by Phay et al. [33]. In 2008, Matsuo et al. [8] identified two loss-of-function heterozygous mutations in *SLC2A9* that caused RHUC by decreased urate reabsorption on both apical and basolateral sides of the proximal renal tubules. To date, 18 patients with EIAKI caused by *SLC2A9* mutation have been reported, most of them have homozygous or compound heterozygous mutations (72.2%, 13/18 and 22.2%, 4/18, respectively) (Table 3).

**Table 3:** Case reports of patients with EIAKI caused by *SLC2A9* mutations

Case reports	Gender	Age(years)	S-UA(mg/dl)	FE-UA(%)	Position	Aminoc acid	Ethnic group	Status	Complications
Dinour [11] 2010	M	46	0.2	> 150	c.224 T > G	L75R	Israeli-Arab	homozygous	EIAKI
	M	24	0.2	> 150	c.224 T > G	L75R	Israeli-Arab	homozygous	EIAKI
	M	19	0.1	> 150	c.224.T > G	L75R	Israeli-Arab	homozygous	EIAKI
Shima [21] 2011	F	11	0.1	58.3	#/dupExonla-11	G207X/#	Japanese	compound heterozygous	EIAKI, PRES
Stiburkova [2] 2013	M	12	0.5	45.8	c.646G > A	G216R	Anglo-Saxon	homozygous	EIAKI
	M	14	0.67	93	c.646G > A/c.968A > G	G216R/N333S	Anglo-Saxon	compound heterozygous	EIAKI
Shen [34] 2014	F	12.1	0.05	> 150	g.68G > A	W23X	China	homozygous	EIAKI
Jeannin [6] 2014	M	24	0.1	> 150	c.1138C > T/c.646G > A	R380W/G216R	Pakistani	compound heterozygous	EIAKI
Androvitsanea [35] 2015	M	15	0.2	> 150	delExon8-12	#	Caucasian	homozygous	EIAKI
Mou [22] 2015	M	11	0.1	139.4	c.1215 + 1G > A	#	China	homozygous	EIAKI, PRES
Windpessl [9] 2016	M	16	0.1	> 150	c.512G > A	R171H	Caucasian	homozygous	EIAKI
Zhou [36] 2016	M	32	0.08	74	c.68C > T/c.857C > T	W23X/W286X	China	compound heterozygous	EIAKI
Claverie-Martin [37] 2018	M	22	1.2	57	c.374C > T	T125M	Spanish	homozygous	EIAKI
	M	12	< 0.5	80	c.374C > T	T125M	Spanish	homozygous	EIAKI
Wang [32] 2019	M	35	0.3	> 150	c.857G > A	W286X	China	homozygous	EIAKI
Wang [38] 2021	M	8	1.9	12.7	c.595-2_595-linsC	#	China	heterozygous	EIAKI
Maalouli [39] 2021	M	20	0.2	119	c.646G > A	G216R	Pakistani	homozygous	EIAKI
Kaynar [40] 2021	M	47	0.1/1.8/0.08	142	c.1419 + 2 T > G	#	Turkey	homozygous	EIAKI

#The literature does not provide; *EIAKI* Exercise-induced acute kidney injury, *PRES* Posterior reversible encephalopathy syndrome

Patients should be advised to avoid strenuous exercise and ensure an adequate intake of fluids after physical activity. On another note, a study involving five participants who took 300 mg of allopurinol daily for five consecutive days and underwent physical fitness testing (PFT) revealed that allopurinol could prevent EIAKI and exercise-induced UA excretion [41]. Furthermore, Frank et al. [42] and Sasaki et al. [43] successfully treated three patients with UA kidney stones and renal hypouricemia using allopurinol, resulting in stone dissolution and preventing further stone formation.

In the present case, the patient was shown to carry two heterozygous mutations in the GLUT9 gene, including a length of 146 bp CNV (chr4:9,982,216–9,982,361) in exon 5 and c.944G > A (p.Trp315Stop) nonsense mutation in exon 7. Although these mutations have not been reported in previous genetic studies on RHUC, they are very likely the cause that leads to the deficiency in urate reabsorption in our patients. CNV is a form of structural variation, corresponding to relatively large regions of the genome that have been deleted or duplicated on certain chromosomes. For example, in this case, 146 bp heterozygous deletion variations were found in exon 5 in the GLUT9 gene. Moreover, DNA sequencing of *SLC2A9* identified a novel heterozygous nonsense mutation, c.944G > A in exon 7 (p.Trp315Stop), which may result in prematurely truncated GLUT9 protein in the patient. If combined these heterozygous mutations with the patient’s clinical feature, it overwhelmingly likely led to the malfunction of the GLUT9 protein. Because other family members were unavailable for the genetic study, we couldn’t get a conclusion that these heterozygous mutations were compound heterozygous mutations. Because this patient’s parents and sister do not have clinical hypouricemia, according to the autosomal recessive mode of inheritance, we speculated that this patient had a compound heterozygous mutation in the GLUT9 gene. These mutations have not been published to date.

A limitation of this case is the lack of the genetic test results from his family members.

This is a report of a patient with RHUC2 due to the mutation of *SLC2A9*, which encodes GLUT9. In clinical practice, the diagnosis of RHUC should be considered in patients manifesting symptoms of recurrent AKI and decreased or normal serum concentrations of UA, particularly after strenuous exercise.

## Data Availability

All data collected from this patient were obtained from Huashan Hospital and are available in this paper.

## Abbreviations

RHUC: Hereditary renal hypouricemia
UA: Uric acid
EIAKI: Exercise-induced acute kidney injury
PRES: Posterior reversible encephalopathy syndrome
BUN: Blood urea nitrogen
Scr: Serum creatinine
FE-UA: Fractional excretion of urate
CK: Creatine kinase
LDH: Lactate dehydrogenase
ANA: Antinuclear antibody
dsDNA: Double-stranded DNA
ANCA: Antineutrophil cytoplasmic antibody
GBM: Glomerular basement membrane
URAT1: Urate transporter 1
GLUT9: Glucose transporter 9
PFT: Physical fitness testing
